# Une dermatophytie en “rosette”

**DOI:** 10.11604/pamj.2016.24.303.3781

**Published:** 2016-08-10

**Authors:** Hafsae Bounniyt, Badredine Hassam

**Affiliations:** 1 Service de Dermatologie Vénérologie, CHU Ibn Sina, Faculté de Médecine et de Pharmacie, Rabat, Maroc

**Keywords:** Champignons kératinophiles, queue du sourcil, alopécie du cuir chevelu, Keratinophilic fungi, tail of the eyebrow, alopecia of the scalp

## Image en médecine

Les dermatophyties sont des affections causées par des champignons kératinophiles. Nous rapportons le cas d'une enfant de 8 ans, bien vaccinée, qui présente depuis 3mois des lésions vésiculo-érosives très prurigineuses regroupées en rosette au niveau de la queue du sourcil gauche (A), des ailes du nez, des faces d'extension des avant bras, et de la partie haute du thorax et du dos, laissant place à des cicatrices hypochromiques (B), sans signes digestifs, par ailleurs l'examen des phanères a révélé une alopécie du cuir chevelu (C) avec des cheveux coupés à leur émergences qui selon la maman évolue depuis une année. Les lésions cutanées évoquaient en premier lieu une dermatite herpétiforme, mais devant la présence de la plaque alopécique, et vu que la biopsie cutanée est un geste invasif pour l'enfant, on a réalisé un examen mycologique qui était en faveur d'un parasitisme pilaire et cutané par le Microsporum Canis. Un traitement à base de Griséofulvine topique et systémique à dose de 20mg/kg/jr pendant 6 semaines a été instauré avec très bonne évolution clinique. Les dermatophyties sont des affections causées par des champignons filamenteux microscopiques Kératinophiles, cliniquement les lésions cutanées réalisent typiquement des placards arrondis ou polycycliques, uniques ou multiples avec une bordure très évocatrice érythémato-vésiculo-squameuse d'évolution centrifuge avec guérison centrale. L'originalité de notre observation réside dans la présentation clinique atypique en rosette.

**Figure 1:**
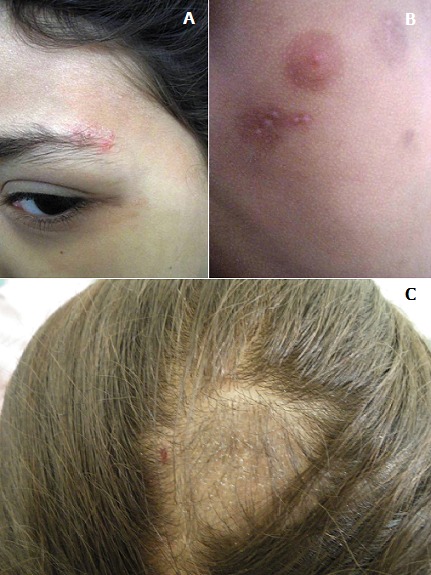
A) lésions vésiculo-érosives en rosette; B) lésions hypochromiques cicatricielles; C) aspect clinique de teigne microsporique

